# Stretchable Magneto-Mechanical Configurations with High Magnetic Sensitivity Based on “Gel-Type” Soft Rubber for Intelligent Applications

**DOI:** 10.3390/gels10010080

**Published:** 2024-01-21

**Authors:** Vineet Kumar, Sang-Shin Park

**Affiliations:** School of Mechanical Engineering, Yeungnam University, 280 Daehak-Ro, Gyeongsan 38541, Gyeongbuk, Republic of Korea; vineetfri@gmail.com

**Keywords:** gel-type soft, silicone rubber, magneto-rheological elastomers, energy harvesting, barium titanate

## Abstract

“Gel-type” soft and stretchable magneto-mechanical composites made of silicone rubber and iron particles are in focus because of their high magnetic sensitivity, and intelligence perspective. The “intelligence” mentioned here is related to the “smartness” of these magneto-rheological elastomers (MREs) to tune the “mechanical stiffness” and “output voltage” in energy-harvesting applications by switching magnetic fields. Hence, this work develops “gel-type” soft composites based on rubber reinforced with iron particles in a hybrid with piezoelectric fillers such as barium titanate. A further aspect of the work relies on studying the mechanical stability of intelligence and the stretchability of the composites. For example, the stretchability was 105% (control), and higher for 158% (60 per 100 parts of rubber (phr) of barium titanate, BaTiO_3_), 149% (60 phr of electrolyte iron particles, EIP), and 148% (60 phr of BaTiO_3_ + EIP hybrid). Then, the magneto-mechanical aspect will be investigated to explore the magnetic sensitivity of these “gel-type” soft composites with a change in mechanical stiffness under a magnetic field. For example, the anisotropic effect was 14.3% (60 phr of EIP), and 4.4% (60 phr of hybrid). Finally, energy harvesting was performed. For example, the isotropic samples exhibit ~20 mV (60 phr of BaTiO_3_), ~5.4 mV (60 phr of EIP), and ~3.7 mV (60 phr of hybrid). However, the anisotropic samples exhibit ~5.6 mV (60 phr of EIP), and ~8.8 mV (60 phr of hybrid). In the end, the composites prepared have three configurations, namely one with electro-mechanical aspects, another with magnetic sensitivity, and a third with both features. Overall, the experimental outcomes will make fabricated composites useful for different intelligent and stretchable applications.

## 1. Introduction

Magneto-rheological elastomers (MREs) are generally termed “smart materials” that originated from changing their mechanical stiffness under magnetic switching tests [1]. These MREs are made up of a “gel-type” soft elastomeric matrix typically from the rubber family. Moreover, iron particles (IPs) are added to the matrix to obtain magnetic sensitivity as well, sometimes, as reinforcing filler to obtain improved mechanical stiffness [2]. Such MREs are well known for their intelligent applications [3,4,5]. These are—(a) by controlling the strength of the magnetic field in MREs, the stiffness tuning makes them useful for dampers and isolators for automobiles; (b) MREs can be useful by integrating them into seals and joints which show mechanical responses to the external conditions; (c) due to the flexibility and softness of MREs, they are excellent candidates for robotics, wearable electronics, and flexible devices; (d) the MREs can be useful for designing smart orthopedic devices that can be applied to adaptive braces, implants and cushions; (e) the tuning stiffness properties of MREs under magnetic switching make them useful for civil engineering applications such as resilience structures against seismic or mechanical loads; and (f) the energy generation in MREs devices in which the mechanical vibrations or dynamic cyclic loadings are converted into useful voltage generation. Overall, the key factor for MREs lies in their ability to undergo reversible changes in their mechanical stiffness, making them useful for various engineering applications [6]. 

Among the intelligent applications of MREs mentioned above, energy harvesting is of the utmost importance to provide voltage for portable devices. These MRE-based devices are useful for the army and other military energy aids where there is no electricity in forests and remote areas. The types of energy harvesting can follow the piezo-electric or triboelectric principles [7]. The main mechanism involves the polarization of the substrate under continuous mechanical deformation. This polarization seems to occur from dipoles originating from piezoelectric materials. These piezoelectric materials are different types like lead-zirconate titanate, barium titanate, etc. [8]. Finally, voltage is generated that can be captured and stored by a capacitor or battery. Such generated energy has various engineering applications such as energy for remote areas with no power supply, vibration damping, and structural health monitoring. Overall, the change in mechanical stiffness under magnetic switching is one of the key reasons for making MREs an attractive candidate for generating energy. Such energy can be stored using capacitors or batteries depending on the nature of materials used during the composite preparations. Moreover, Wu et al. [9] provide intelligent sensing functions for smart hydrogel skins that are stretchable, tough, and transparent. These intelligent functions are enabled by machine learning and are generally termed “smart materials”, such as MREs. 

It is noticeable that the selection of the right materials remains the key factor in influencing the magnetic sensitivity, response rate, energy generation, efficiency, and output voltage in MREs [10]. Generally, the MREs used for energy generation and magnetic sensitivity consist of piezoelectric material, iron particles, and stretchable polymer mostly from the elastomer family. In some cases, the conductivity of the substrate of the device can be improved by adding conductive fillers like carbon nanotube or graphene [11]. The conductive filler along with the piezoelectric material remains a topic of recent research [11,12]. Furthermore, the iron particles are added to improve the magnetic sensitivity in MREs. These iron particles have different morphologies, sizes, and a high density. Generally, the most frequently used are carbonyl iron particles with a spherical shape, size in microns, and density of around 7 g/cm^3^ [13]. The high density of these iron particles remains a matter of concern, but generally settles down with time often in magnetic fluids. In some cases, it is also challenging to control this settling problem in MREs during solution or latex mixing. However, it is not as severe as in magnetic fluids. Nevertheless, a well-optimized methodology for preparing MREs is required to disperse these iron particles uniformly [14]. Finally, the type of elastomer matrix plays a vital role in influencing the final properties of the MREs. The frequently used elastomer matrix used in MREs is natural rubber, styrene-butadiene rubber, or silicone rubber [15,16]. Mostly, silicone rubber is favored and the first choice of the researchers working on MREs. This is because of its easy processing, simple vulcanization process, lower hardness, good aging properties, and finally better resistance against acidic conditions [16,17]. These constituents directly influence the final properties of the MREs. 

As already discussed, the mechanical stiffness can be controlled by the type of ingredients and magnetic switching tests. The magnetic field magnitude influences this smart property in MREs, and viscosity controlled by adding thinner is also possible to control mechanical stiffness [18]. The addition of thinner strongly reduces the mechanical stiffness, making the MREs “Gel-type” soft and useful for ultra-soft applications such as energy harvesting, soft robotics, wearable electronics, and stretchability [18]. Thus, the present work explores the addition of thinner in silicone rubber matrix to tune its stiffness to “Gel-type” soft composites with a hardness below 15. The different types of constituents like barium titanate as a piezoelectric material are used for improving the output voltage for energy harvesting. Then, the electrolyte iron particles make the MREs magnetic-sensitive with a high response force and good magnetic sensitivity. Tadesse et al. [19] reviewed the hydrogels with soft nature that are extremely useful for various soft applications such as supercapacitors for wearable electronics. Similarly, “Gel-type” soft silicone rubber was used as an elastomeric matrix for soft engineering applications such as self-powered wearable materials. The further novelty of this work in relation to the existing literature can be referred to in Table 1 below.

## 2. Results and Discussion

### 2.1. Mechanical Properties under Static Compression Mode

**Figure 1:**
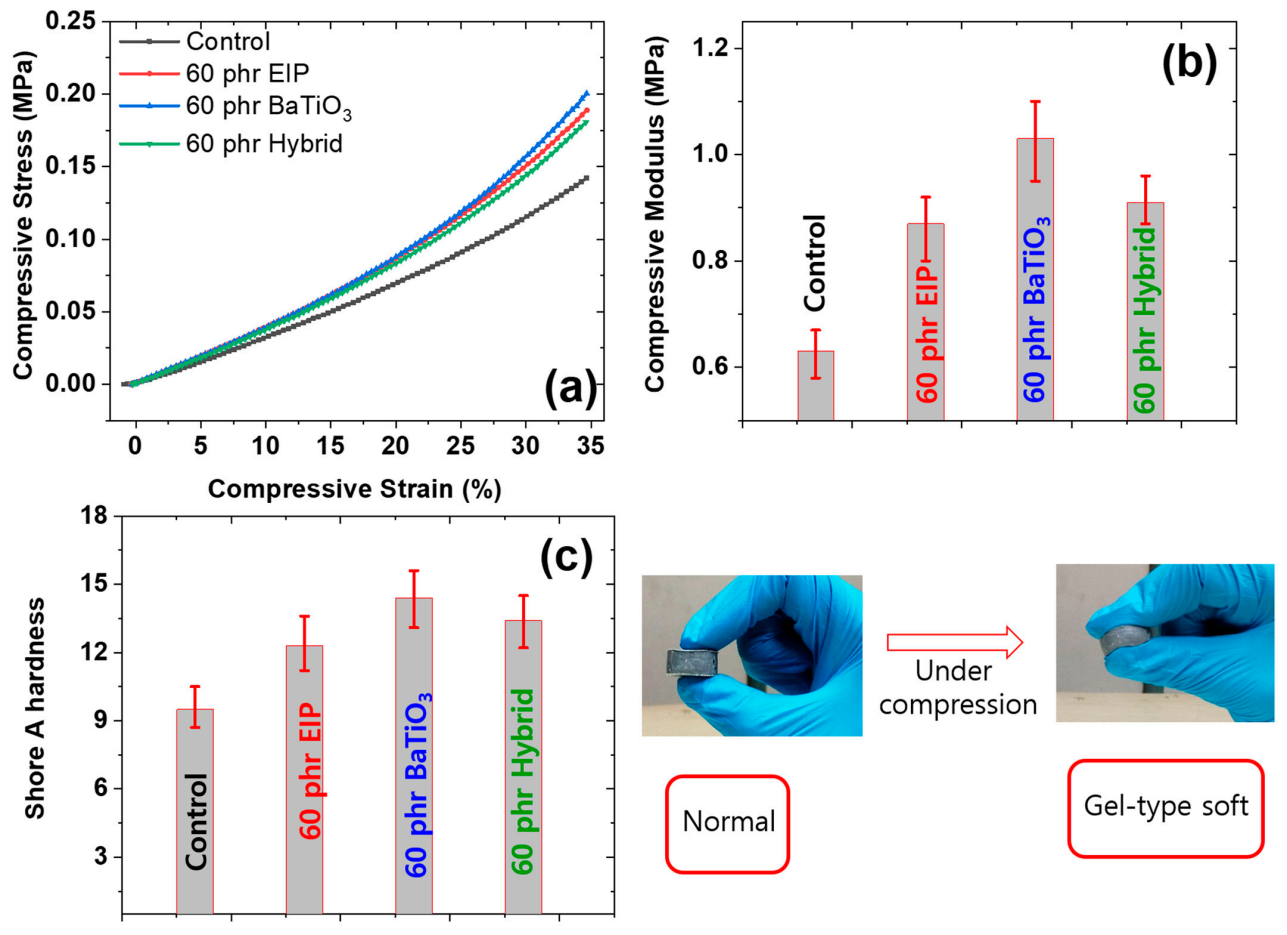
(**a**) stress–strain of different formulations; (**b**) compressive modulus of samples; (**c**) shore A hardness of the composites.

It is well documented that the mechanical stiffness in MREs can be controlled by switching magnetic fields [25,26]. Here, the stiffness was tuned by adding thinner (lowering the viscosity), making the elastomer “gel-type” soft. This is useful for soft applications such as energy harvesting and wearable electronics. Figure 1a shows the stress–-strain of composites by increasing the compressive strain. The results show that the compressive stress was improved with an increase under compression. It can be accounted for through an increase in packing fractions of the microstructures in the composite [27]. The packing fraction generally refers to the ratio of the volume of EIPs or BaTiO_3_ or hybrid filler to the total volume of the composite material. The packing fraction can be low, medium, or high depending on the content of the filler and the extent of the compression. The lower packing fraction results when the compression value is low or when the filler content is low. However, the higher packing fraction can be witnessed at higher filler loadings or compression values. Zhu et al. [28] evaluate the packing properties of the filler particles and their secondary particles for dental resins. They conclude that mechanical properties such as flexural strength, flexural modulus, and compressive strength are significantly affected by the packing properties of fillers and other secondary particles in the resin composite. Moreover, the higher packing fraction or a low packing fraction is less relevant for a higher magnetic response or magnetic effect. Therefore, it can be optimized to a level where the MRE response can be maximized. In the present work, the 60 phr of filler was optimized from our previous studies, which provide the best comprehensive mechanical stiffness [25,29]. For example, a lower filler content to 60 phr comes under low packing fractions while a higher filler loading to 60 phr leads to a higher packing fraction or aggregation of fillers in the SR matrix. Moreover, the ability to influence the mechanical properties in real time makes the MREs valuable for different engineering applications such as vibration control systems and soft robotics.

**Scheme 1:**
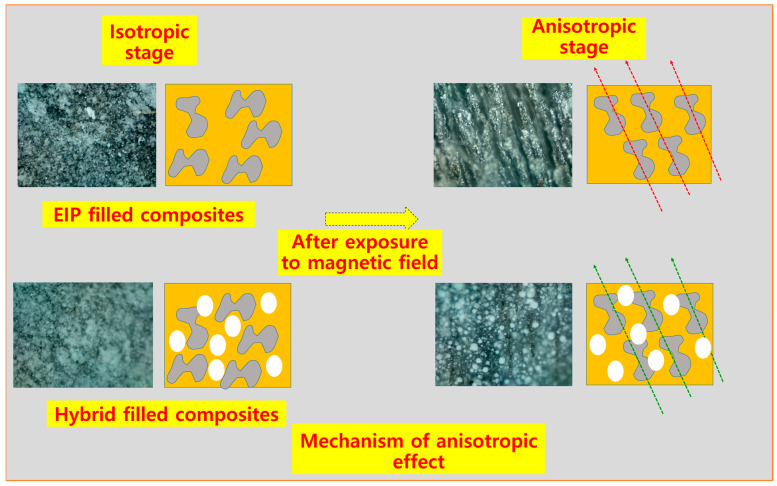
Isotropic and anisotropic effects and the mechanism behind such effects.

Figure 1b shows the compressive modulus of different formulations of MREs. The results show that the overall modulus was significantly affected by the type of filler and the addition of thinner. Among all the composites prepared, the BaTiO_3_ was found to be effective for enhancing stiffness while the control sample came out to be the lowest modulus. However, the EIPs containing samples were also lower while the hybrid was the optimum with a balanced stiffness along with magnetic sensitivity. Overall, the EIP-filled composites exhibit great magnetic effect but a lower stiffness while BaTiO_3_ shows good energy harvesting but no magnetic sensitivity. Therefore, the hybrid filler should be selected as a key material that possesses good energy harvesting along with magnetic active material. Moreover, the elastic modulus can be further altered by magnetic switching which can influence the stiffness. This magnetic response is typically reversible, allowing a tunable stiffness [30]. This concept is illustrated in detail in Scheme 1 in the coming section. 

**Figure 2:**
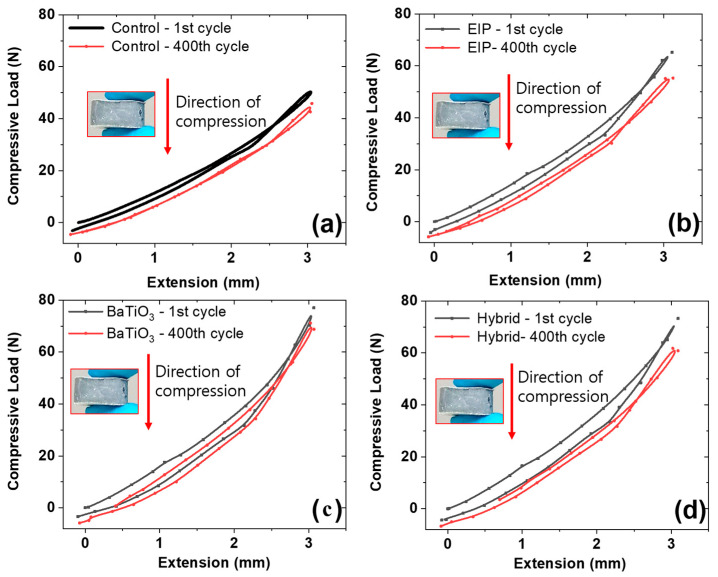
Load-extension curves under compression cycling (**a**) control; (**b**) EIP; (**c**) BaTiO_3_; (**d**) Hybrid.

The purpose of studying the hardness in Figure 1c was to understand the degree of softness in the composite materials and the influence of different fillers on the hardness of such composites [31]. Various advantages of lower viscosity help in achieving the lower hardness as targeted in the present study. These are—(a) achieving a significant tunable mechanical stiffness with magnetic switching. For example, if the viscosity of the sample is lower, there is a chance of a high anisotropic effect, which is the main aim of this study. (b) The lower hardness of the MREs renders them more adaptable for use in soft applications such as tissue engineering, medical implants, soft robotics, and wearable electronics, thereby making them multifunctional materials. (c) Softness in MREs may reduce the potential for damage or more stability against mechanical failure, thereby making the devices based on them more durable with efficient fatigue properties. Moreover, the present study also confirms that the composites are “gel-type” soft materials that exhibit tuning stiffness along with useful energy harvesting, especially in hybrid-filled composites. As expected, the hardness was lower than 15, making these samples soft with a higher compressibility, good elastic range, and lower viscosity, which are useful for soft applications. These soft MREs allow a better tuning in stiffness through the use of magnetic switching and the addition of thinner to lower the viscosity of the composites. This process makes them suitable for engineering applications that need high flexibility, wearable devices, and a versatile mechanical and magnetic response. The soft samples with low viscosity often exhibit lower hysteresis losses. This means that the subjected material can be easily deformed and recovered efficiently, making them useful for high-durability applications. This process can be understood more clearly in Figure 2. However, some limitations of such soft MREs are the limited load-bearing capacity and complex modeling and design [32]. Overall, researchers will continue to explore ways to study new and novel designs of MREs that can examine the optimized properties as per the application in need. 

### 2.2. Mechanical Properties under Compression Cycling

Studying the mechanical aspects of compression under the cyclic mode is essential for designing MREs for various engineering applications. These applications include dampers, vibration isolators, and other adaptive structures [33]. Experimental techniques like multi-hysteresis are often used to understand such dissipation loss phenomena and thus are presented in Figure 2. The multi-hysteresis involves applying and releasing the external load repeatedly. Moreover, the dissipated energy under loading–unloading cycles is studied and understood in Figure 2 for different samples. Firstly, compression cycling provides information on the ability of the material to absorb and release heat energy during multi-hysteresis and is crucial for damping applications. Figure 2a shows the lowest dissipation losses while BaTiO_3_ and hybrid-filled composites show higher dissipation losses than EIP-filled composites. However, the magnitude of dissipation losses is not as significant for the control sample as for the filled composites. Moreover, the resilience property was better for the control sample than other filled samples. This was supposed to be a lower viscosity and higher elasticity of the control sample than those from filled composites [34]. Understanding the resilience property is essential in MREs, especially in applications where repeated deformations are performed. Moreover, it is also useful to investigate the permanent deformations that remain after the removal of the mechanical deformations in the cyclic mode. This feature is useful for understanding the material’s tendency to undergo permanent deformation. In present studies, all the filled composites are shown to possess higher permanent deformation as compared to the control sample.

In addition to the multi-hysteresis and dissipation losses, the change in compression load between the 1st and 400th cycle was important and is presented in Figure 2a–d. The load-displacement profiles show that the resilience property was best in the control samples even at the 400th cycle compared to other filled composites. Moreover, evaluating the dissipation losses from the 1st to the 400th cycle under compression cycling is important in terms of energy efficiency, and minimum dissipation losses are desired for best performance [35]. Finally, it was found that the dissipation losses were higher for the 1st cycle than the 400th cycle for all composites. This feature can be hypothesized to break down fresh interfacial interactions in the 1st cycle. However, successively at the 400th cycle, these interfacial interactions stabilize thereby leading to stable dissipation losses. Overall, such mechanical aspects are important for understanding the behavior of a particular material. These results help in designing and optimizing MREs for an application of interest.

### 2.3. Mechanical Properties under Static Tensile Mode

**Figure 3:**
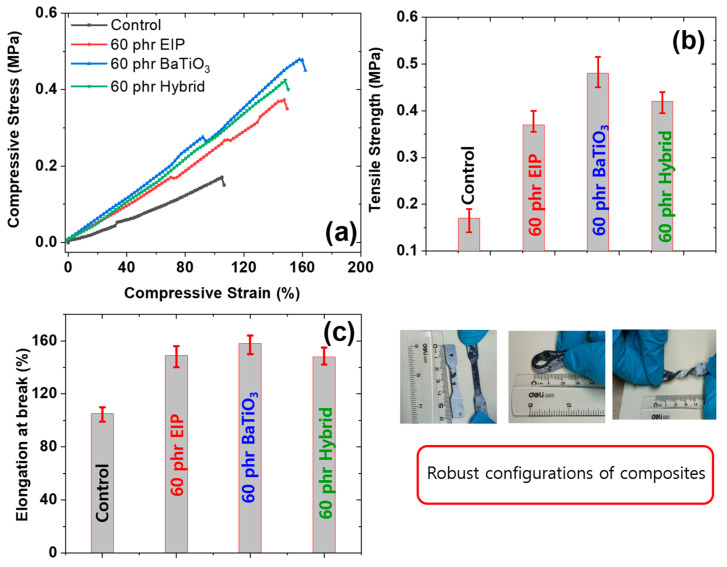
(**a**) stress–strain of different formulations; (**b**) tensile strength of samples; (**c**) elongation at break of the composites.

Studying and tuning the mechanical stiffness of MREs through thinner or magnetic switching is of great interest. Generally, the addition of thinner makes the composite “gel-type” soft by reducing the viscosity of the elastomer matrix used in MREs. Moreover, the nature of the strain (compressive or tensile) under which these properties are studied is fascinating [36]. Thus, stress–strain under tensile mode was performed and studied in Figure 3a. The results show that stress increases almost linearly with increasing tensile strain. It is proposed due to interfacial interactions within the compound and in-rubber microstructures that oppose the applied strain [37]. Moreover, it is proposed that the value of the stress is quite low, making it “gel-type” soft as noticed in the hardness values in Figure 1c above. This was proposed by the addition of thinner that renders the composites soft, making them useful for soft electronic applications such as wearable electronics, adaptive structures, and damping systems. Moreover, the influence of filler type on the output voltage in energy harvesting is an important factor in rubber composites. However, the extent of the increase concerning the type and content of the particular filler is a matter of interest and has thus been studied. Here, the compressive stress increases with the filler type and is best for BaTiO_3_ as a filler and lowest for EIP-based fillers in the SR matrix. This could be proposed due to the “nano-effect” of BaTiO_3_ that exhibits a higher interfacial area, and better filler dispersion in the SR matrix. These properties also influence the tensile strength and elongation at break and are discussed in brief in Figure 3b,c below. Overall, the behavior of MREs can be tuned by selecting the right materials for fabrication, type, and content of iron particles, as well as the magnetic field magnitude based on the target application.

Figure 3b and Figure 3c show the tensile strength and elongation at break for different compounds, respectively. The results show that both of them are correlated and consistent with the other mechanical properties like Figure 1 obtained in this work. For example, the properties are best for BaTiO_3_-based composites, following hybrid-filled ones, while the EIP and control sample shows lower mechanical properties. Since the concentration of all fillers is the same, the particle size is lower for BaTiO_3_ and higher for the EIP-filled SR matrix. The larger particle size of the EIP leads to a lower reinforcing effect due to a poor interfacial area and thus a poor stress transfer among microstructures in the SR matrix [38]. Moreover, the structural design including the arrangement and dispersion of filler particles and their geometry can influence the mechanical performance of the composites presented in the present work. Overall, the reinforcing effect of particulate fillers is crucial for optimizing their response to external deformations [39]. These are the magnitude of strain or tailoring the properties as needed in a particular material. For example, the thinner was added in the present work to lower the stiffness while it was further controlled by magnetic switching tests in samples containing iron particles.

### 2.4. Isotropic—Anisotropic Mechanical Properties under Compression

**Figure 4:**
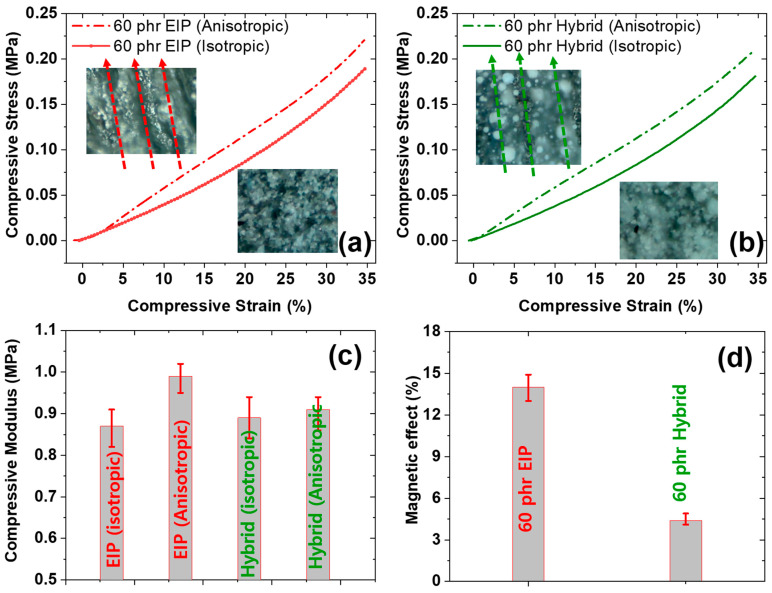
Stress-strain of isotropic and anisotropic composites—(**a**) EIP; (**b**) hybrid filler; (**c**) compressive modulus of samples; (**d**) magnetic effect of the composites.

Firstly, the isotropic are the composites that may or may not contain iron particles and fillers that are dispersed randomly within the SR matrix. In isotropic composites, the mechanical and magnetic responses are identical, regardless of the direction of mechanical deformation. However, anisotropics are those samples that must contain iron particles in single or hybrid form and are subject to the alignment of fillers under a magnetic field [40]. Hence, the properties of anisotropic materials depend upon the direction of alignment of iron particles concerning the direction of external deformations. As discussed earlier, MREs mainly exhibit anisotropy in which the stiffness can be controlled by magnetic switching tests. Figure 4a,b presents the results obtained for isotropic and anisotropic materials under compression. The stress-strain curves show that the stress was higher in anisotropic samples for both EIP and hybrid filler-based composites. The higher mechanical performance for anisotropic composites is due to the alignment of the EIP particles in the vertical direction to the applied strain. Overall, the distinction between isotropic and anisotropic samples is fundamental in understanding how the alignment of iron particles varies with a particular application of interest [41]. 

Figure 4c provides the details of the influence of anisotropy on the compressive modulus of composites. As discussed in Figure 1, the packing fraction of the filler particles plays a dominating role in influencing the modulus of different materials. In the context of MREs, the packing fraction can have a dominating role in influencing output properties, as witnessed in Figure 4c. The anisotropic effect of the modulus is often related to the alignment of EIPs in the SR matrix. The packing fraction of the present composites is optimum due to the optimum content of EIP and hybrid fillers in the SR matrix. However, this optimum packing fraction shifts to a higher order depending upon the magnitude of the magnetic field and type of iron particles such as EIPs in the present work. Such an increase in the packing fraction results in a greater stiffness of the samples. This increase in stiffness improves the compressive modulus, which is higher for anisotropic composites. Such an anisotropic stiffening effect is desirable where control of the specific alignment is crucial [42,43]. It can be understood more effectively in Figure 4d. A higher anisotropic effect for EIP-filled composites was witnessed while it was lower for hybrid-filled composites. There is a saturation effect in the packing fraction of fillers under a magnetic field. This saturation point was faster because of the presence of BaTiO_3_ particles causing saturation in addition to EIP particles in a hybrid-filled composite. Another reason was the lower EIP content in the hybrid filler (30 phr) than with the EIP as only filler (60 phr). This content results in a lower alignment effect of EIPs in the hybrid filler than with the EIP as the only filler. Overall, understanding of the packing fraction and anisotropy is critical for optimizing the performance of these smart material-based MREs.

### 2.5. Mechanism of Anisotropic Effect

The anisotropic effect generally refers to the change in stiffness of the composites due to the alignment of the EIP in the direction of the magnetic field. The mechanism of the anisotropic effect involves various key points [44,45]. Some of them are (a) the MREs containing a “gel-type” soft SR matrix filled with magneto-sensitive EIPs. The SR provides robust flexibility and stretchability, and allows the EIPs to align in a magnetic environment; (b) the distribution of the EIPs can be random and described as “isotropic materials” or aligned in a particular required direction and termed as “anisotropic materials”; they are presented clearly in Scheme 1 above. The fundamental principle in such an alignment of EIPs resulted from the magnetic torque exhibited by EIPs under a magnetic field that forces them to align in the magnetic field direction; (c) such an anisotropic effect results from the change in stiffness, modulus, and damping properties. The extent of such changes depends upon the EIP concentration and strength of the magnetic field. Overall, the anisotropic effect in MREs arises from the alignment of EIPs in the direction of the magnetic field. This property allows the controlled modulation of the behavior of MREs for in-demand desired properties. 

### 2.6. Isotropic—Anisotropic Properties under Compression

As stated already, the anisotropic properties refer to the directional dependencies in their mechanical stiffness. These occurred due to the alignment of iron particles in the direction of magnetic exposure, thereby leading to a change in stiffness. Moreover, the study of the anisotropic material with loading-unloading cycles is important to establish their influence under compressive mechanical deformation [46]. 

**Figure 5:**
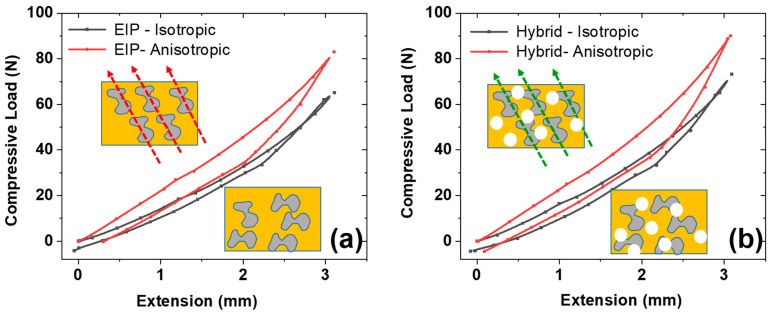
Load-extension curves of isotropic and anisotropic compounds under compression cycles (**a**) EIP; (**b**) Hybrid.

Thus, Figure 5a,b shows the behavior of load-extension curves for EIPs and hybrid-filled MREs, respectively. The results show that in both cases, the dissipation losses were higher for anisotropic samples than isotropic ones. This dissipation energy is associated with the damping properties of the MREs and it influences the applications such as vibration control and other resilience properties. It can be proposed that the dissipation losses are random in isotropic samples due to the uniform distribution of EIPs and BaTiO_3_ particles in MREs. However, the dissipation losses are direction-oriented in anisotropic composites. This is due to the alignment of EIP particles in a particular direction as presented in Scheme 1 above. The dissipation losses in anisotropic samples result in higher energy losses in the direction of alignment. This results in increased damping properties in a specific direction. It also us helps in understanding that the dissipation losses can be tailored by controlling the EIPs alignment as per the required application of interest. Overall, the dissipation energy in MREs can be influenced by the internal structures of the compound [47]. These internal structures can be isotropic or anisotropic, as described in Scheme 1. The choice of these two types of structures in MREs can be preferred depending on the required applications.

## 3. Application as Energy Harvesting

### 3.1. Isotropic Energy Harvesting under Compression Cycling

**Figure 6:**
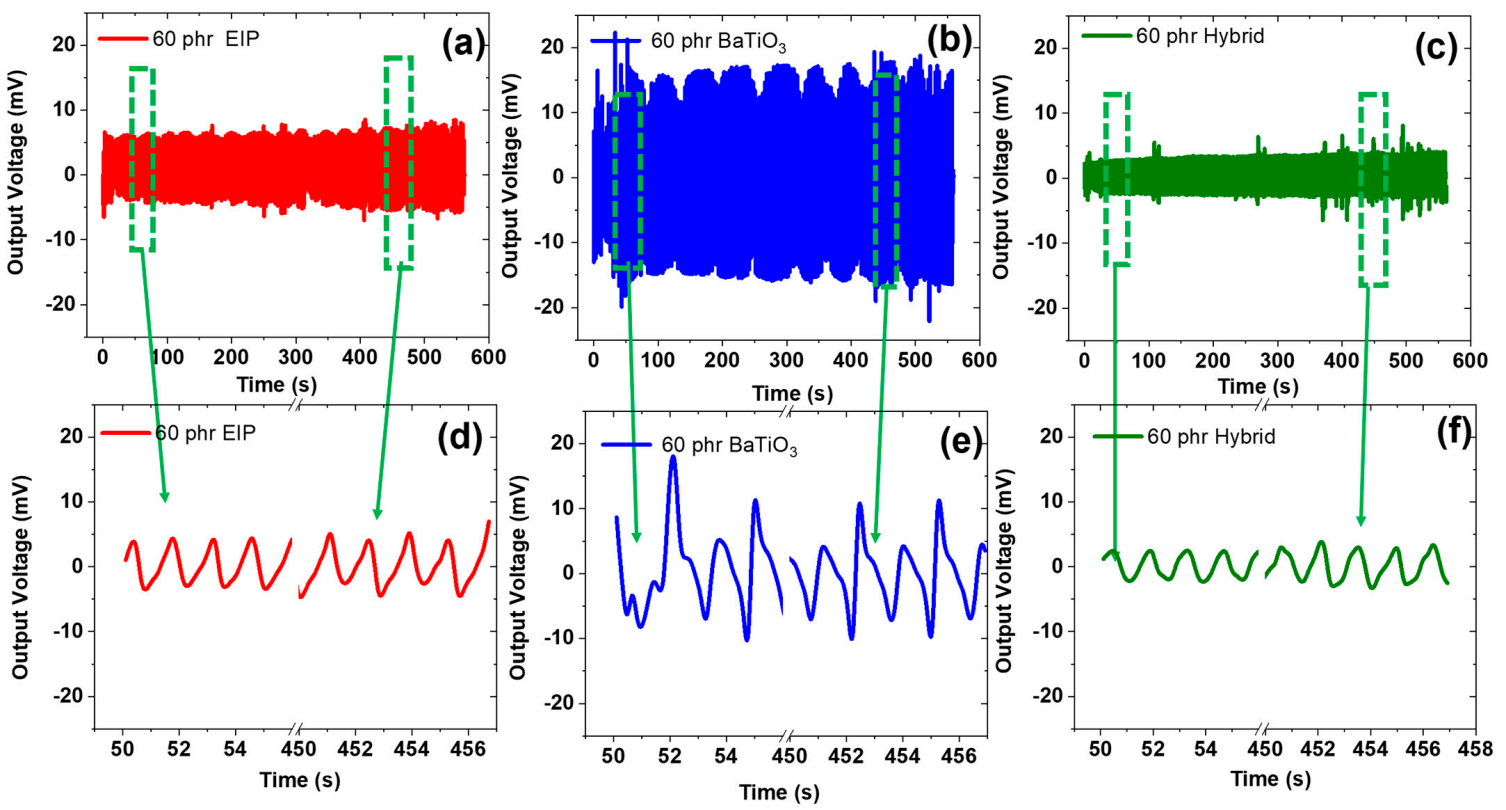
Isotropic energy harvesting for different formulations: (**a**,**d**) EIP; (**b,e**) BaTiO_3_, and (**c,f**) Hybrid.

The classification of energy generation for isotropic composites shows the voltage generation uniformly in all directions of MREs. This was credited to the uniform dispersion of EIPs and BaTiO_3_ in isotropic samples. Figure 6a–f shows the dependency of output voltage concerning the nature of the material used during fabrication. As expected, the energy harvesting was best for piezoelectric materials like BaTiO_3_-based MREs (Figure 6b). The higher output voltage for BaTiO_3_-based MREs is due to the generation of a large number of charged particles under cycling loading. These charged particles cause polarization of the substrate. Finally, the copper electrodes are charged with these displaced charged particles towards the electrodes [48]. This eventually leads to the generation of a higher output voltage, as desired. Moreover, the lower voltage generation for EIPs and hybrid-filled composites was noted and presented in Figure 6a,c. The lower voltage in these samples could be due to poor structural design owing to the large particle size of the EIP and its poor electrical conductivity. This leads to a lower polarization effect in the substrate, thereby resulting in a lower output voltage in such MREs. However, Figure 6d–f shows that the voltage stability was better for EIPs and hybrid-filled systems than BaTiO_3_-based MREs. This behavior makes them better candidates for applications that require optimum output voltage and high durability. However, voltage stability is not common in MREs as these smart materials are mostly limited to a change in stiffness under magnetic sensitivity. Yet, the hybrid-filled system that contains both BaTiO_3_ and EIPs is most useful as it exhibits good energy generation in addition to better magnetic sensitivity, magnetic response rate, and optimum durability. Overall, isotropic energy harvesting technologies are of key interest for various soft applications such as wearable electronics, structural health monitoring, or energy generation from vibrant environments [49].

### 3.2. Anisotropic Effect in Energy Harvesting under Compression Cycling

**Figure 7:**
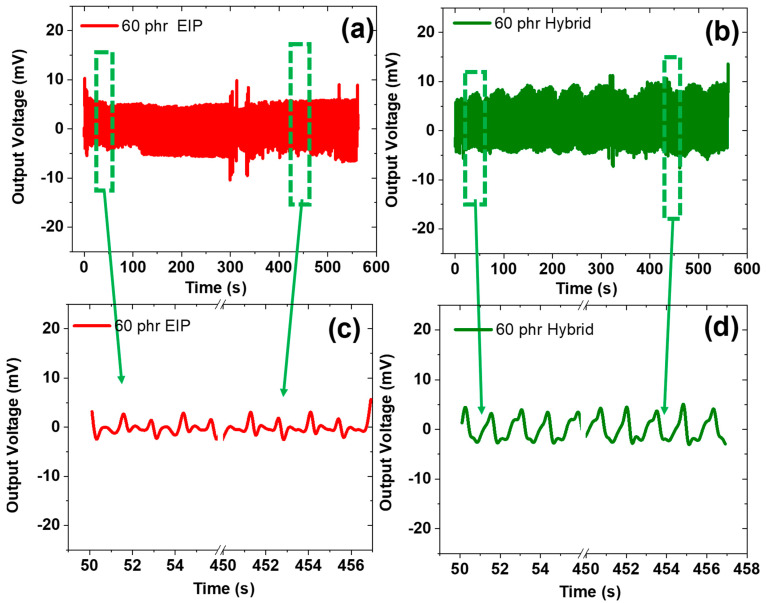
Anisotropic energy generation for different composites: (**a**,**c**) EIP; (**b,d**) Hybrid.

As discussed already, the anisotropic effect in energy harvesting refers to the voltage generation in a particular direction. For example, the anisotropic materials exhibit different energy conversion or response features in the direction of alignment as reported in Figure 7a,b. The energy generation is proposed to be higher than in isotropic materials, especially in hybrid materials containing piezoelectric materials and EIPs in MREs (Figure 7a). Due to the alignment of EIPs in hybrid fillers, the response force of EIPs also forces the BaTiO_3_ particles to realign roughly. It therefore influences the overall voltage generation. It can be seen in Scheme 1 that some BaTiO_3_ particles tend to re-align, leading to higher voltage generation in anisotropic hybrid-filled composites. Moreover, anisotropic piezoelectric materials exhibit different coefficients along the crystallographic directions. This process affects the efficiency of energy conversion when the material is subjected to external mechanical deformations [50]. Moreover, the voltage generation in EIP-filled anisotropic composites is not much affected as compared to isotropic samples due to the poor electrical properties of the EIPs in the SR matrix. Overall, the understanding of anisotropy in energy harvesting could be crucial to enhanced energy generation. Also, the optimization of the anisotropy in the required way may lead to a robust voltage output in such MREs [51]. Such optimization involves the selection of the right materials, the right parameters of fabrication, and the optimum magnetic field in a specific direction as required.

## 4. Conclusions

This work addresses the MREs made from a “gel-type” soft matrix containing EIP or BaTiO_3_ or a hybrid of both fillers. The results show the promising use of such composites for soft applications such as robotics or other biomedical uses that require soft and intelligent materials. These soft MREs show the influence of mechanical stiffness under magnetic switching or the addition of thinner in the SR matrix. These MREs are characterized by various mechanical properties such as modulus, stretchability, or tensile strength. For example, the tensile strength was 0.17 MPa (control), and higher for 0.48 MPa (60 phr of BaTiO_3_), 0.37 MPa (60 phr of EIP), and 0.42 MPa (60 phr of hybrid). The magnetic properties such as the anisotropic effect on the mechanical stiffness tuning under magnetic switching are also addressed. For example, the anisotropic effect was 14.3% (60 phr of EIP) and 4.4% (60 phr of hybrid). The energy harvesting for both isotropic and anisotropic composites was also established. For example, the energy harvesting for isotropic samples exhibits ~20 mV (60 phr of BaTiO_3_), ~5.4 mV (60 phr of EIP) and ~3.7 mV (60 phr of hybrid). Moreover, the anisotropic samples exhibit ~5.6 mV (60 phr of EIP) and ~8.8 mV (60 phr of hybrid). Overall, the combining of these improved composites with novel and optimized processing conditions are important and in focus. Finally, these optimizations not only help in the uniform dispersion of EIPs and BaTiO_3_ but are also useful in creating sensing mechanisms. The versatility of these configurations was useful for various engineering applications such as soft robotics or energy harvesting.

## 5. Materials and Methods

### 5.1. Materials

The MREs were prepared by using transparent silicone rubber (SR) (KE-441-KT). This rubber can be vulcanized at room temperature after adding a hardener (CAT-RM). It was a condensation-type vulcanizing SR system. The viscosity of the SR used in the present work was 15 Pa-s. Moreover, the thinner was used to lower the viscosity of the SR matrix, making the samples mechanically soft. The RTV silicone oil with a diluent effect and no organic solvent like toluene was used as a thinner. The SR, thinner, and hardener were purchased from the Shin-Etsu Chemical Corporation Ltd., Japan. The piezoelectric material used to enhance output voltage during energy harvesting was BaTiO_3_ (US3833, 100 nm, 99.9%, cubic, US Research Nanomaterials Inc., Houston, TX, USA). The magnetic response and sensitivity of the composites were enhanced by adding electrolyte-type iron particles (Fe#400, 8–10 µm, 98.8% iron, ~7–8 g/cm^3^, Aometal Corporation Ltd., Gomin-si, Korea). The morphological features and XRD of both fillers are presented in Figure S1 (Supporting Information). Finally, the mold-releasing agent was purchased from Nabakem, Korea.

### 5.2. Methods Used in Characterizations of MREs

**Scheme 2:**
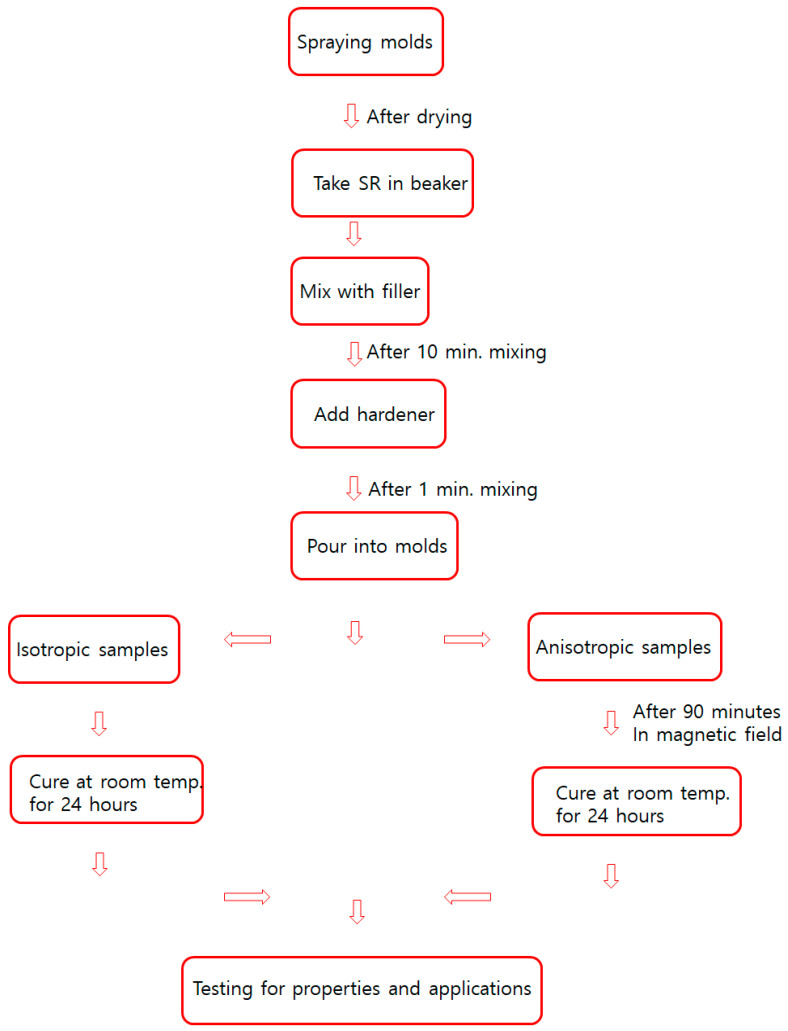
Steps for preparation of the composites.

The dispersion of EIP, BaTiO_3_, and hybrid filler in the SR matrix was studied by optical images. For that, the 0.5 mm thick sample of both isotropic and anisotropic composites was investigated under an optical microscope (Sometech Inc., Seoul, Republic of Korea). The mechanical stiffness was tested under a universal testing machine (UTS, Lloyd Instruments, Bognor Regis, UK). These mechanical properties were observed at compressive and tensile strain. The cylindrical samples (20 × 10 mm^2^) were used for observing the compressive modulus while dumbbell samples (25 × 10 × 2 mm^3^) were used for observing the tensile strength and fracture strain. The strain rate was 4mm/min for compressive mode experiments and 200 mm/min for tensile mode experiments. Both these experiments were conducted under a load cell of 1 kN. The preload was 0.1 N for compressive and 0.5 N mm for tensile tests. All these mechanical tests were carried out following the guidelines of DIN 53 504 standards. The anisotropic samples were prepared by keeping the just prepared “gel-type” cylindrical samples containing EIP and hybrid fillers inside the magnetic field at 1Tesla for 90 min at 25 °C. Finally, these samples were taken out of the magnetic field and kept at 25 °C for 24 h for curing. These complete steps in the preparation of isotropic and anisotropic samples are presented in Scheme 2.

### 5.3. Preparation of MREs

The steps for preparation of the MREs were optimized in our previous study [25]. The steps are summarized in Scheme 2 below and the formulation of the composites are presented in Table 2.

## Figures and Tables

**Table 1:** The novelty of the present work over the existing literature is described below.

S. No.	Rubber Type	Filler Type	Anisotropic Effect	Energy Harvesting	Reference
1.	Silicone rubber	Iron particles with different size	10%	-	[20]
2.	Polydimethylsiloxane	Carbonyl iron particles	107.87%	-	[21]
3.	Polydimethylsiloxane	Carbonyl iron particles	-	-	[22]
4.	Polydimethylsiloxane	Carbonyl iron particles	−14.2%	-	[23]
5.	Silicone rubber and silicone oil	Carbonyl iron particles	118%	-	[24]
6.	Silicone rubber and thinner	Electrolyte iron particles and barium titanate	~14.3%	~5.4 mV for isotropic and ~5.6 mV for anisotropic sample	This work

**Table 2:** The formulations * of these composites are described below.

ID for Samples	SR Content (phr)	BaTiO_3_ Content (phr)	EIP Content (phr)	BaTiO_3_ + EIP Content (phr)	Thinner Content (phr)	Vulcanizing Agent Content (phr)
Control	100	-	-	-	20	2
BaTiO_3_—composites	100	60	-	-	20	2
EIP—composites	100	-	60	-	20	2
Hybrid—composites	100	-	-	30 + 30	20	2

* These formulations and filler content are optimized and adapted from previous work [25,29].

## Data Availability

All data and materials are available on request from the corresponding author. The data are not publicly available due to ongoing researches using a part of the data.

## Supplementary Materials

The following supporting information can be downloaded at: https://www.mdpi.com/article/10.3390/gels10010080/s1, The morphological features and XRD of both fillers are presented in Figure S1.
