# Supplementary material for: Stretchable Magneto-Mechanical Configurations with High Magnetic Sensitivity Based on “Gel-Type” Soft Rubber for Intelligent Applications
Source: Gels. 2024 Jan 21;10(1):80. doi: 10.3390/gels10010080 (PMC10815761; doi:10.3390/gels10010080)
Supplement: Supplementary file 1 [file gels-10-00080-s001.zip › gels-2831835-supplementary.pdf]

gels

MDPI

Supplementary Materials

Stretchable Magneto-Mechanical Configurations with High magnetic Sensitivity Based on "Gel-Type" Soft Rubber for Intelligent Applications

Vineet Kumar and Sang-Shin Park *

School of Mechanical Engineering, Yeungnam University, 280 Daehak-Ro, Gyeongsan 38541, Gyeongbuk, Republic of Korea; vineetfri@gmail.com
* Correspondence: pss@ynu.ac.kr

Results and discussion

SEM and XRD of the filler particles

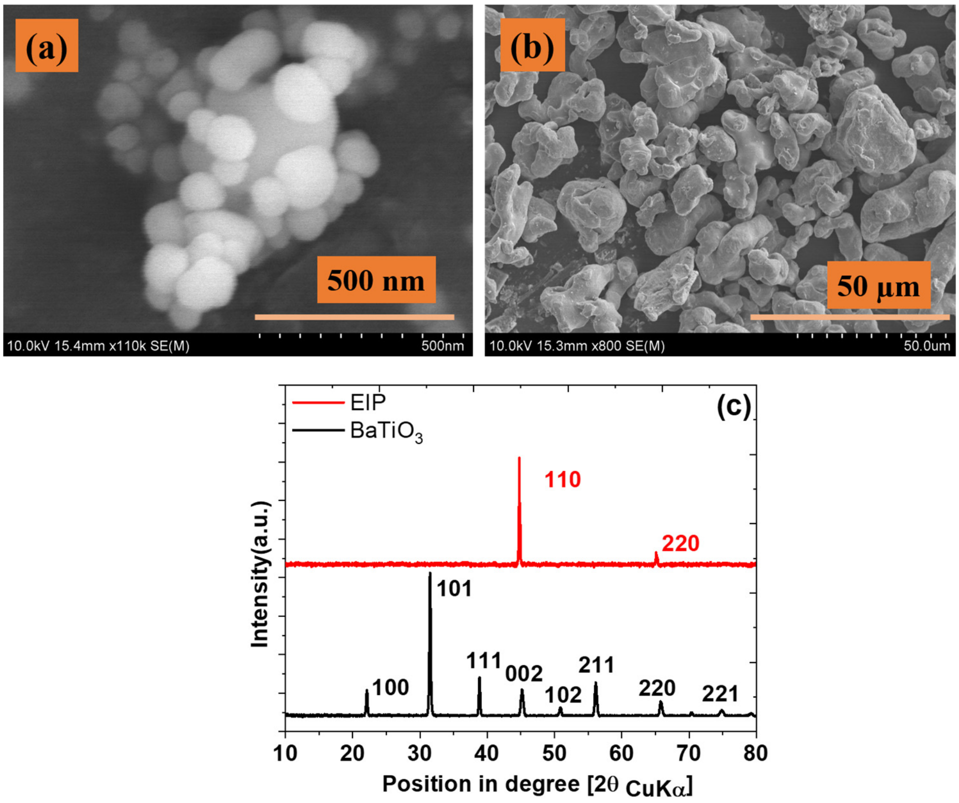

Figure S1. SEM of the filler particles: (a) $BaTiO_3$; (b) EIP; (c) XRD of both fillers.
